# Lipopolysaccharide-mediated signal transduction: Stabilization of TNF-alpha mRNA contributes to increased lipopolysaccharide-stimulated TNF-alpha production by Kupffer cells after chronic ethanol feeding

**DOI:** 10.1186/1476-5926-2-S1-S31

**Published:** 2004-01-14

**Authors:** Raj Kishore, Megan R McMullen, Enzo Cocuzzi, Laura E Nagy

**Affiliations:** 1 Department of Nutrition, Case Western Reserve University, Cleveland OH, 44106-4906, USA

## Introduction

Alcoholic liver disease (ALD) develops in approximately 20% of all alcoholics with a higher prevalence in females [1]. The development of fibrosis and cirrhosis is a complex process involving both parenchymal and non-parenchymal cells resident in the liver, as well as the recruitment of additional cell types to the liver in response to damage and inflammation [2]. Kupffer cells, the resident macrophages in the liver, are critical to the onset of ethanol-induced liver injury. Ablation of Kupffer cells prevents the development of fatty liver and inflammation, early events in the progression of ethanol-induced liver damage, in rats chronically exposed to ethanol via intragastric feeding [3]. Endotoxin (or lipopolysaccharide (LPS)), a component of the cell wall of gram-negative bacteria, is an important activator of Kupffer cells, stimulating the production of inflammatory and fibrogenic cytokines, as well as reactive oxygen species. LPS concentration is increased in the blood of alcoholics [4,5] and rats exposed to ethanol via intra-gastric infusion [6], probably due to impaired barrier function of the intestinal mucosa [7]). In a series of elegant experiments using transgenic animals from the laboratory of Ron Thurman, a working model for the development of alcoholic liver disease has been developed. This model proposes that increased exposure of Kupffer cells to LPS during chronic ethanol consumption results in increased production of inflammatory mediators, in particular TNF-alpha and reactive oxygen species, leading to the progression of fatty liver, inflammation and fibrosis, characteristic of ALD [7]. In addition to this increased exposure of Kupffer cells to LPS in response to ethanol, we and others have shown that chronic ethanol also sensitizes Kupffer cell responses to LPS [8,9]. We hypothesize that increased sensitivity to LPS stimulation after chronic ethanol exposure likely contributes to the progression of liver injury.

### Role of TNF-alpha in the progression of alcoholic liver disease

TNF-alpha is thought to play a particularly critical role in the pathogenesis of ALD. TNF-alpha is one of the principal mediators of the inflammatory response in mammals, transducing differential signals that regulate cellular activation and proliferation, cytotoxicity and apoptosis [10,11] In addition to its role in acute septic shock, TNF-alpha has been implicated in the pathogenesis of a wide variety of inflammatory diseases [11,12,14] as well as in the progression of alcoholic liver disease [7,15] The role of TNF-alpha in the development of ethanol-induced liver injury has been well characterized in animal models [7,15]

Production of TNF-alpha is one of the earliest responses of the liver to injury [15]. Circulating TNF-alpha is increased in the blood of alcoholics and in animals chronically exposed to ethanol [16,17]. Antibiotic treatment decreases TNF-alpha expression and ethanol-induced liver injury in rats exposed to ethanol via intra-gastric infusion [7], suggesting that increased TNF-alpha after ethanol exposure is due, at least in part, to increased exposure to LPS. In addition to increasing LPS exposure, chronic ethanol also increases sensitivity to LPS. For example, long-term ethanol consumption increases the susceptibility of rats to endotoxin-induced liver injury [8,18]. Moreover, we have shown that LPS-stimulated TNF-alpha secretion is increased in Kupffer cells isolated from rats fed ethanol in their diet for 4 weeks compared to pair-fed controls [9,19,20].

### Regulation of LPS-stimulated TNF-alpha production

**Figure 1:**
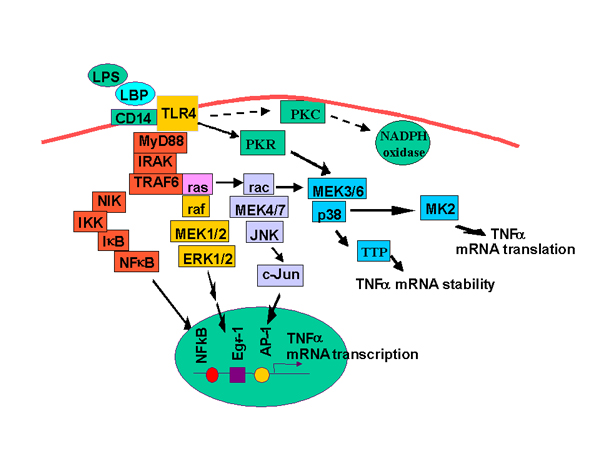
Lipopolysaccharide-stimulated signal transduction pathways which increase TNF-alpha production in macrophages. Schematic representation of some of the signaling intermediates potentially involved in regulation of TNF-alpha expression in macrophages.

Production of inflammatory cytokines is a highly regulated process; regulation has been reported at the level of transcription, translation and secretion [21,22]. Transcriptional activation of TNF-alpha by LPS requires the activation of a distinct set of transcription factors binding to at least two regions of the TNF-alpha promoter which include NF kappa B, Egr-1 and AP-1 binding sites [23] (Figure 1) [24]. While the exact array of transcription factors interacting with the TNF-alpha promoter is to some extent cell and species specific [25], recruitment of NF kappa B and early growth response 1 protein (Egr-1), as well as increased c-jun binding to a CRE/AP-1 site, appear to be required for full activation of TNF-alpha expression in most types of macrophages [23,24]. Activation of each of these nuclear transcription factors is mediated by specific LPS-mediated signaling cascades (Figure 1). LPS binds to a cell surface receptor, CD14, which, via interactions with the toll-like receptor 4 (TLR4) [26], stimulates a complex array of signal transduction cascades [27,28] Stimulation of macrophages with LPS activates tyrosine kinases, protein kinase C, nuclear factor kappa B (NF kappa B), as well as members of the mitogen activated protein kinase family, including ERK1/2 (extracellular receptor activated kinases 1/2), p38 and c-jun N-terminal kinase (JNK) [27].

Ethanol disrupts a number of hormone and neurotransmitter dependent signaling pathways [29], including many of the same signaling pathways activated by LPS in macrophages. In a series of recent experiments, we have found that chronic ethanol feeding disrupts specific LPS-stimulated signal transduction pathways which regulate both TNF-alpha transcription and mRNA stability in Kupffer cells [19,20,30]. Chronic ethanol had complex effects on the regulation of LPS-stimulated TNF-alpha mRNA transcription; the transcriptional activity of NF kappa B was dramatically decreased, but this was compensated for by increased Egr-1 activity [19,30]. Despite these complex changes, chronic ethanol exposure had no net effect on the rate of TNF-alpha transcription [20]. Therefore, we hypothesized that increased LPS-stimulated TNF-alpha mRNA accumulation in Kupffer cells isolated from rats chronically exposed to ethanol might be due to a stabilization of TNF-alpha mRNA.

## Methods

### Chronic ethanol feeding protocol

Male Wistar rats (150 g) were allowed free access to liquid diet [31] with 17% of calories as ethanol for 2 days and then provided with diet containing 35% of the calories from ethanol for 4 weeks. Controls were pair-fed a liquid diet that was identical to the ethanol diet except that maltose dextrins were isocalorically substituted for ethanol [32]. Procedures involving animals were approved by the Institutional Animal Care Board at Case Western Reserve University.

### Kupffer cell isolation and culture

Kupffer cells were isolated as previously described [9] except that CMRL media was used to isolate and culture Kupffer cells. Briefly, livers were perfused with 0.05% collagenase and the resulting suspension of liver cells treated with 0.02% pronase for 15 min at 12 degrees C. The cell suspension was centrifuged 3 times at 50 – g for 2 min and the supernatant collected after each centrifugation. The pooled supernatant was then centrifuged at 500 – g for 7 min to collect non-parenchymal cells. Kupffer cells were then purified by centrifugal elutriation [9].

Isolated Kupffer cells were suspended in CMRL with 10% fetal bovine serum and penicillin-streptomycin at a concentration of 2 – 10^6 ^cells/ml and plated onto 96 well (0.2 ml/well), 6-well (3 ml/well) or 100 mm plates (5 ml/well) culture plates. After 2 h, non-adherent cells were removed by aspiration and fresh media supplied. Assays were initiated after 20–24 h in culture.

### Culture and chronic ethanol exposure of RAW264.7 macrophages

The mouse macrophage-like RAW264.7 cell line was obtained from American Type Culture Collection (ATCC, Rockville, MD). Cells were cultured in DMEM supplemented with 10% FBS and penicillin-streptomycin at 37 degrees C in a 5% CO_2 _atmosphere. For ethanol treatments, cells were incubated with 25 mM ethanol for 48 h; culture dishes were wrapped in parafilm to minimize the evaporation during culture. Culture dishes with untreated cells were also wrapped in parafilm.

### Ribonuclease protection assay for TNF-alpha mRNA

After 20 h in culture, Kupffer cell media was removed and replaced with fresh media containing 10% fetal bovine serum. Cells were then stimulated with 0 or 100 ng/ml LPS for 1 h. In some experiments, cells were pre-incubated with PD98059, SB20380 or vehicle (DMSO) for 2 h prior to LPS stimulation, or treated with 5 micrograms/ml actinomycin D 1 h after LPS stimulation and then harvested after 1–2 h. Total RNA was isolated by the TRIzol method (Gibco, Grand Island, NY). Rat cytokine multiprobe DNA templates (Pharmingen, San Diego, CA) were used to synthesize *in vitro *transcribed anti-sense riboprobes and ribonuclease protection assays were carried out following manufacturer's instructions. Samples were run on 5% sequencing gels, dried and autoradiographed.

## Results and Discussion

### Chronic ethanol increases LPS-stimulated TNF– expression

Kupffer cells isolated from rats fed ethanol for 4 weeks accumulated 2-fold higher concentration of TNF-alpha in their media in response to challenge with LPS, with no difference in TNF-alpha accumulation between the pair- and ethanol-fed groups when cells were not treated with LPS (basal) [9,19]. Increased accumulation of bioactive TNF-alpha after chronic ethanol was associated with increased accumulation of TNF-alpha mRNA in response to LPS [19,20].

Increased LPS-stimulated TNF alpha production by Kupffer cells after chronic ethanol feeding could be due to a direct effect of chronic ethanol exposure on the Kupffer cell itself or to a more systemic response, involving other tissues and organs sensitive to ethanol. If chronic ethanol acts directly on the Kupffer cell, then exposure of macrophages to ethanol during in vitro culture should mimic the *in vivo *response. RAW264.7 macrophages were cultured with and without 25 mM ethanol for 48 h and then stimulated or not with 100 ng/ml LPS for 2 to 4 h. While LPS increased TNF-alpha production in control cells, secretion of TNF-alpha in response to LPS was increased by 1.7–2.2-fold in cells cultured with ethanol compared to controls [30]. Increased LPS-stimulated TNF-alpha secretion after chronic ethanol exposure was associated with increased TNF-alpha mRNA accumulation [30].

**Figure 2:**
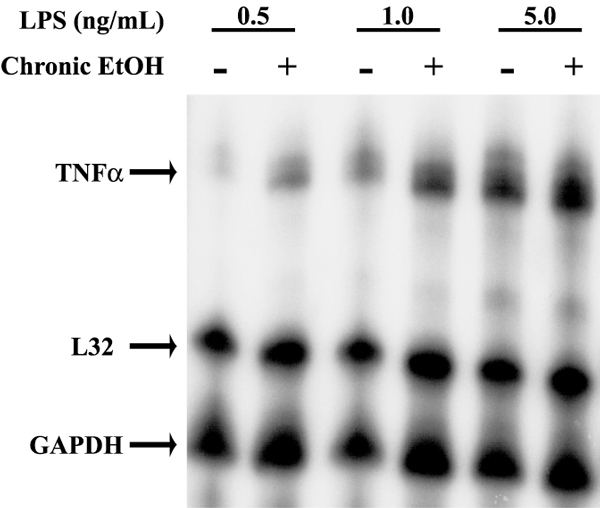
Chronic ethanol exposure sensitizes RAW 264.7 macrophages to low concentrations of lipopolysaccharide. RAW 264.7 macrophages were cultured with and without 25 mM ethanol for 48 h and then stimulated with 0–5 ng/ml lipopolysaccharide (*E. coli *serotype O26:B6) in the presence of 10% serum for 60 min. RNA was isolated and quantity of TNF-alpha and GAPDH mRNA measured by ribonuclease protection assay.

Chronic ethanol exposure increased the sensitivity of RAW 264.7 macrophages and Kupffer cells to LPS, rather than the maximal response to LPS. Enhanced TNF-alpha accumulation after chronic ethanol feeding was observed at low concentrations of LPS [We have found that the efficacy of LPS to stimulate TNF-alpha mRNA varies between lots of LPS purchased from the same source (unpublished observations).], but was not different from pair-fed at higher concentrations of LPS [9]. Similarly, while chronic ethanol exposure of RAW 264.7 macrophages enhanced TNF-alpha mRNA accumulation at lower concentrations of LPS over controls (Figure 2), at higher concentrations there was no longer a difference between control and ethanol treated cells (Figure 2).

Taken together, these data indicate that there is a strong similarity between the effects of ethanol feeding on Kupffer cell responses to LPS and the in vitro response of RAW 264.7 macrophages to culture with chronic ethanol. These results suggest that changes in TNF-alpha production by macrophages observed after long term ethanol exposure in vivo are not due solely to systemic responses, such as increased exposure to endotoxin/LPS [4-6] or changes in retinoic acid status [5,6,33], but are due, at least in part, to a direct effect of ethanol exposure on macrophage function.

### LPS- induced TNF-alpha mRNA is stabilized by chronic ethanol exposure

While LPS-induced TNF-alpha production is controlled at transcriptional, post-transcriptional and post-translational levels [21], increased transcription is the initial response to LPS. Therefore, we investigated whether chronic ethanol affected LPS-stimulated TNF-alpha transcription. Kupffer cells isolated from pair- and ethanol-fed rats, as well as RAW 264.7 macrophages cultured with or without 25 mM ethanol, were stimulated with 0 or 100 ng/ml LPS, nuclei isolated and used for run-on transcription assays. We found that chronic ethanol exposure, either *in vivo *or during culture, had no net effect on TNF-alpha transcription [20].

Since chronic ethanol was not acting to increase transcription of TNF-alpha, we hypothesized that chronic ethanol-induced increases in LPS-stimulated TNF-alpha mRNA accumulation might reflect an increased half-life of the transcripts. To test this hypothesis, mRNA stability experiments were performed in Kupffer cells and in RAW264.7 macrophages. Kupffer cells from ethanol- and pair-fed rats were stimulated with 0 or 100 ng/ml of LPS for 60 min. Cells were further incubated in the presence or absence of actinomycin D for 1–2 h. RNA was isolated and TNF-alpha mRNA expression measured by ribonuclease protection assays [20]. Chronic ethanol consumption stabilized LPS-induced TNF-alpha mRNA in Kupffer cells isolated from ethanol-fed rats (t_1/2 _> 100 min), compared to those isolated from pair-fed rats (t_1/2 _&lt; 40 min). A similar effect of chronic ethanol was observed on the TNF-alpha mRNA stability in RAW 264.7 macrophages. In control cells, LPS-induced TNF-alpha mRNA decayed with an approximate half-life of 35 min. However, treatment of cells with 25 mM ethanol for 48 h not only increased the accumulation of TNF-alpha mRNA, but also substantially stabilized the TNF-alpha transcript (t_1/2 _>100 min) [20]. These data demonstrate that exposure to chronic ethanol both *in vivo *and *in vitro *results in a marked stabilization of LPS-induced TNF-alpha mRNA.

### Inhibition of p38 MAP kinase specifically eliminates ethanol-mediated stabilization of TNF-alpha mRNA

Activation of p38 and ERK1/2 MAP kinases has been linked to mRNA stabilization of otherwise short-lived cytokine and other immediate early response genes [34-39]. We have reported that chronic ethanol exposure, both *in vivo *and during culture, increases both LPS-stimulated ERK1/2 and p38 activation [19,20,30]. Therefore, we asked whether ethanol-induced potentiation of p38 or ERK1/2 MAP kinases was involved in the stabilization of TNF-alpha mRNA observed after chronic ethanol exposure. Kupffer cells isolated from ethanol- and pair-fed rats were pretreated with either 20 micromolar SB203580 or 50 micromolar of PD98059 followed by stimulation with LPS. After stimulation with LPS, Kupffer cells were further treated or not with actinomycin D. Total RNA was isolated and TNF-alpha mRNA levels measured by ribonuclease protection assays [20]. Inhibition of p38 activation completely abrogated ethanol-mediated stabilization of TNF-alpha mRNA [20]. In contrast, inhibition of ERK1/2 activation by PD98059 had no effect on ethanol-mediated stabilization of TNF-alpha mRNA [20].

## Conclusions

Chronic ethanol exposure, either by in vivo feeding to rats or *in vitro *during macrophage culture, resulted in a stabilization of the LPS stimulated TNF-alpha mRNA transcripts [20]. Interestingly, Motomura and colleagues [33] recently reported a stabilization of TNF-alpha mRNA transcripts in Kupffer cells isolated from ethanol-fed rats even when they were not stimulated with exogenous LPS. This stabilization was associated with a depletion of retinoic acid during in vivo ethanol exposure [33]. Taken together, these data indicate that chronic ethanol-induced stabilization of TNF-alpha mRNA contributes significantly to increased LPS-stimulated TNF-alpha secretion. Importantly, inhibition of p38 MAPK activity prevented the stabilization of TNF-alpha mRNA and reduced secretion of TNF-alpha in response to LPS [20]. These data suggest that regulation of TNF-alpha mRNA stability mediates increased TNF-alpha production during ethanol consumption and thus contributes to the progression of inflammation during alcoholic liver disease.
